# Treatment with Antibiotics that Interfere with Peptidoglycan Biosynthesis Inhibits Chloroplast Division in the Desmid *Closterium*

**DOI:** 10.1371/journal.pone.0040734

**Published:** 2012-07-17

**Authors:** Hiroko Matsumoto, Katsuaki Takechi, Hiroshi Sato, Susumu Takio, Hiroyoshi Takano

**Affiliations:** 1 Faculty of Science, Kumamoto University, Kurokami, Kumamoto, Japan; 2 Graduate School of Science and Technology, Kumamoto University, Kurokami, Kumamoto, Japan; 3 Center for Marine Environment Studies, Kumamoto University, Kurokami, Kumamoto, Japan; 4 Bioelectrics Research Center, Kumamoto University, Kurokami, Kumamoto, Japan; Duke University Medical Center, United States of America

## Abstract

Charophytes is a green algal group closely related to land plants. We investigated the effects of antibiotics that interfere with peptidoglycan biosynthesis on chloroplast division in the desmid *Closterium peracerosum–strigosum–littorale* complex. To detect cells just after division, we used colchicine, which inhibits *Closterium* cell elongation after division. Although normal *Closterium* cells had two chloroplasts before and after cell division, cells treated with ampicillin, D-cycloserine, or fosfomycin had only one chloroplast after cell division, suggesting that the cells divided without chloroplast division. The antibiotics bacitracin and vancomycin showed no obvious effect. Electron microscopic observation showed that irregular-shaped chloroplasts existed in ampicillin-treated *Closterium* cells. Because antibiotic treatments resulted in the appearance of long cells with irregular chloroplasts and cell death, we counted cell types in the culture. The results suggested that cells with one chloroplast appeared first and then a huge chloroplast was generated that inhibited cell division, causing elongation followed by cell death.

## Introduction

Although it is accepted that the ancestor of primary plastids is an endosymbiont of a free-living cyanobacteria, plastids are thought to have no peptidoglycan layer, except the cyanelles that are peptidoglycan-armed plastids in a group of glaucocystophyte algae [1], [2]. If plastid peptidoglycans have been lost in the green plant lineage, antibiotics that inhibit peptidoglycan biosynthesis would be expected to have no effect on the cells. However, treatment with the β-lactam antibiotic ampicillin results in the appearance of macrochloroplasts in the moss *Physcomitrella patens*, liverwort *Marchantia polymorpha*, and lycophyte of pteridophytes *Selaginella nipponica* (reviewed in [3]). Continuous observation of treated *P. patens* cells revealed that the decrease in the number of chloroplasts resulted from cell division without chloroplast division. On the other hand, no effect of ampicillin was observed on chloroplast number in cultured tomato cells [4].

**Figure 1:**
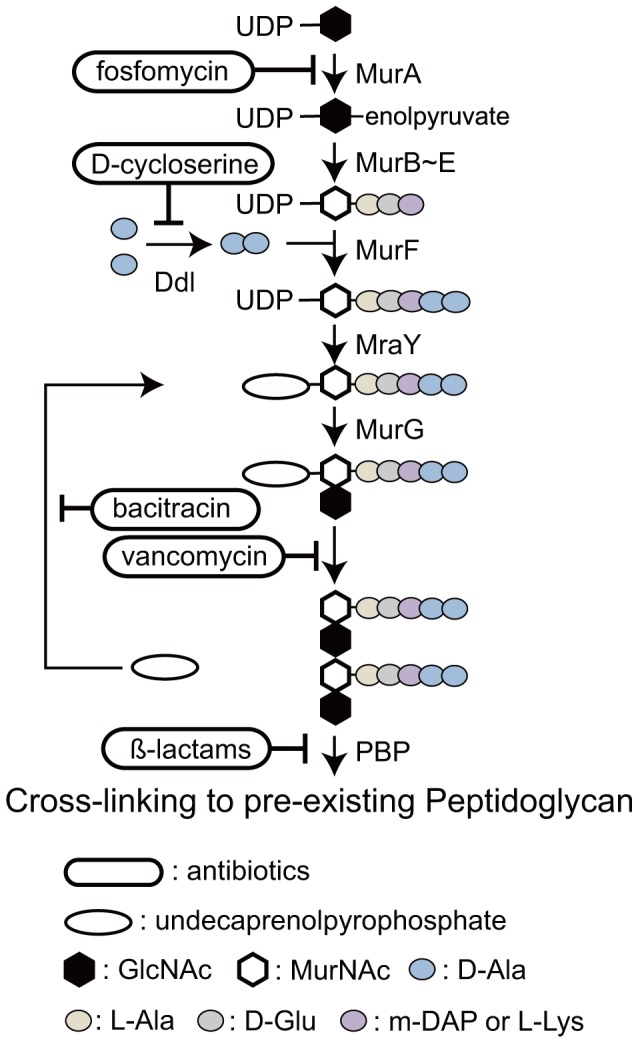
The bacterial peptidoglycan biosynthesis pathway and antibiotics that interfere with it. Starting with the transfer of an enolpyruvate to UDP-GlcNAc by the enzyme MurA, shown beside the pathway, MurB catalyzes the formation of UDP-MurNAc in the cytoplasm. MurC, D, E, and F mediate the formation of UDP-MurNAc- pentapeptide. D-alanyl-D-alanine is synthesized by Ddl. Two enzymes, MraY and MurG, form GlcNAc-MurNAc-pentapeptide-pyrophosphoryl-undecaprenol, which is transferred to the periplasm. This disaccharide pentapeptide monomer unit is cross-linked to pre-existing peptidoglycan by penicillin-binding proteins (PBPs) to form the sacculus surrounding the bacterial cell membrane.

In addition to ampicillin, treatment with D-cycloserine or fosfomycin, which interfere with peptidoglycan synthesis at different steps than β-lactams (Fig. 1), resulted in a macrochloroplast phenotype in *P. patens* cells, suggesting retention of a peptidoglycan system [5]. The genome project for *P. patens* and a search of the expressed sequence tag (EST) database indicated that it apparently has all of the genes necessary for the primary peptidoglycan biosynthesis pathway (reviewed in [3]). Moreover, gene disruption of the *P. patens* (*Pp*) *MurA*, *PpMurE*, *PpMraY*, or *PpPbp* genes in *P. patens* resulted in the appearance of a few macrochloroplasts in each cell, confirming that these *Mur* genes are related to plastid division in the moss [6], [7]. This seems reasonable because bacterial peptidoglycans are related to cell division in addition to protection from osmotic and mechanical lysis.

Genome sequences of seed plants, such as *Arabidopsis thaliana*, rice, grapevine, and Populus, indicate that they have only a few *Mur* genes (reviewed in [3]). On the other hand, the spikemoss of lycophytes *Selaginella moellendorffii* genome [8] suggestes the existence of all the *Mur* genes. Moreover, in addition to ampicillin, fosfomycin treatment resulted in macrochloroplasts in *S. nipponica* [9]. Thus, we proposed that the loss of plastid peptidoglycan occurred during pteridophyte and/or seed plant evolution [3]. The plants derived from the primary endosymbiotic event are the glaucocystophytes, red algae, and green plants. We can identify no *Mur* genes in the genome of the red alga *Cyanidioschyzon merolae* [10], suggesting that red algae have lost the ability to synthesize peptidoglycan. Based on data regarding plant genomes, we proposed that the loss of peptidoglycan occurred independently in the lineage of red algae [3].

Do green algae retain a plastid peptidoglycan system? It is now widely agreed that land plants (embryophytes) evolved from green algae, in particular, streptophyte algae, also referred to as charophycean algae. Charophycean algae are thought to have split from chlorophyte algae about 725–1200 MY ago [11], [12]. The genomic sequences of chlorophyte algae, such as *Chlamydomonas reinhardtii* and *Volvox carteri*, reveal no *Mur* genes, except *MurE*, suggesting the loss of plastid peptidoglycan occurred in the chlorophytes [13], [14].

So, do charophycean algae retain a plastid peptidoglycan system? Because there is no report of the genome sequence of charophycean algae, we do not have access to data on plastid peptidoglycan genes from genome databases. Reports on the effects of antibiotics on bryophytes and pteridophytes suggest that the treatments may be useful for the detection of plastid peptidoglycans. Charophyceans comprise five lineages (orders) of freshwater green algae: Charales, Coleochaetales, Zygnematales, Klebsormidiales, and Chlorokybales. The Charales, Coleochaetales or Zygnematales have been considered to be a sister group of the land plants in the charophycean algae. Recent reports have suggested that the Zygnematales or a clade consisting of Zygnematales and Coleochaetales are most likely the sister group [15]. The desmid *Closterium*, which belongs to Zygnematale, is used generally to investigate sexual reproduction in charophycean algae [16]. The possibility of inhibiting *Closterium* chloroplast division by β-lactams was reported as an unpublished observation in a paper by Iino and Hashimoto [17]. However, the effects of other antibiotics were not mentioned. Thus, we selected the unicellular charophycean alga *Closterium peracerosum-strigosum-littorale* complex (*C*. *psl.* complex) to examine plastid peptidoglycans.

## Results

**Figure 2:**
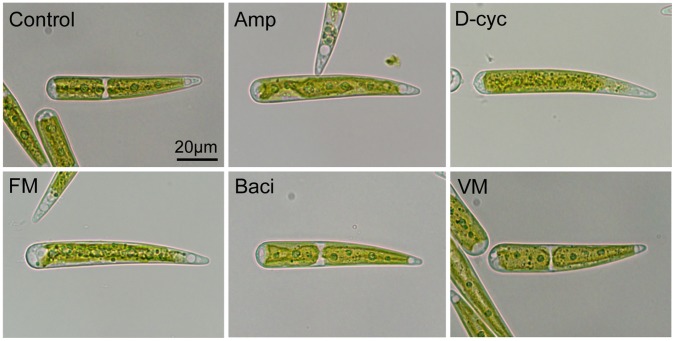
Effects of antibiotics on *Closterium* cells. Addition of colchicine led to production of tadpole-shaped cells by inhibiting cell elongation after division. These cells were easy to distinguish from normal crescent-shaped cells. Without antibiotics (Control), cells had two chloroplasts. When *Closterium* cells were treated with 100 µM ampicillin (Amp), 100 µM D-cycloserine (D-cyc), or 250 µM fosfomycin (FM), cells with only one chloroplast appeared. On the other hand, 100 µM bacitracin (Baci) and vancomycin (VM) did not affect the number of chloroplasts.

To detect the effects of antibiotics that inhibit bacterial peptidoglycan synthesis in chloroplast morphology or division, *Closterium psl.* complex was treated with five antibiotics (Fig. 2). Because treatments with the antibiotics for five days resulted in the appearance of long cells and long dead cells with huge chloroplasts (described later), we used the microtubule-disrupting agent colchicine to detect *Closterium* cells just after division. Division of *Closterium psl.* complex can be synchronized in the dark period under a 16/8 h light/dark cycle [18]. Normal crescent-shaped *Closterium* cells have two chloroplasts. Because cell and chloroplasts divide synchronically, the generated semicells also have two chloroplasts. After cell division, the semicell elongates at the division site to reestablish crescent-shaped morphology. Because treatment with 2 mM colchicine inhibits cell elongation, we could detect the cells that divided in the dark period by cell morphology (Fig. 2). Without antibiotics, almost all (99%) of the cells had two chloroplasts. Treatment with ampicillin, D-cycloserine, or fosfomycin resulted in the appearance of cells with one chloroplast (Fig. 2; 81%, 70%, and 92%, respectively). This suggested that these antibiotics inhibited chloroplast division in *Closterium* cells. In contrast, treatment with bacitracin or vancomycin did not affect the number of chloroplasts in a cell.

To confirm the effects of antibiotics, following a four day treatment with ampicillin, we removed the antibiotics at 2 h after the light to dark shift, and started 2 mM colchicine treatment at the same time. At the end of the dark period, we observed *Closterium* cells through cell division. With continuous treatment with ampicillin, levels of cells with one or two chloroplasts were 72 and 28%, respectively. Similarly, in the medium from which ampicillin was removed, the level of cells with one chloroplast was 76%. The cells were incubated continuously under light conditions for 16 h; in the light period, cell division of *Closterium* does not occur. Moreover, to interfere with cell division, in addition to inhibiting cell elongation, the colchicine concentration was raised to 4 mM according to a published report [19]. At the end of the 16 h light period, the level of cells with two chloroplasts was increased to 72% in the medium without ampicillin, while it was unchanged (28%) in the continuously ampicillin-treated samples. These results demonstrate that inhibition of chloroplast division was directly dependent on the ampicillin treatment.

**Figure 3:**
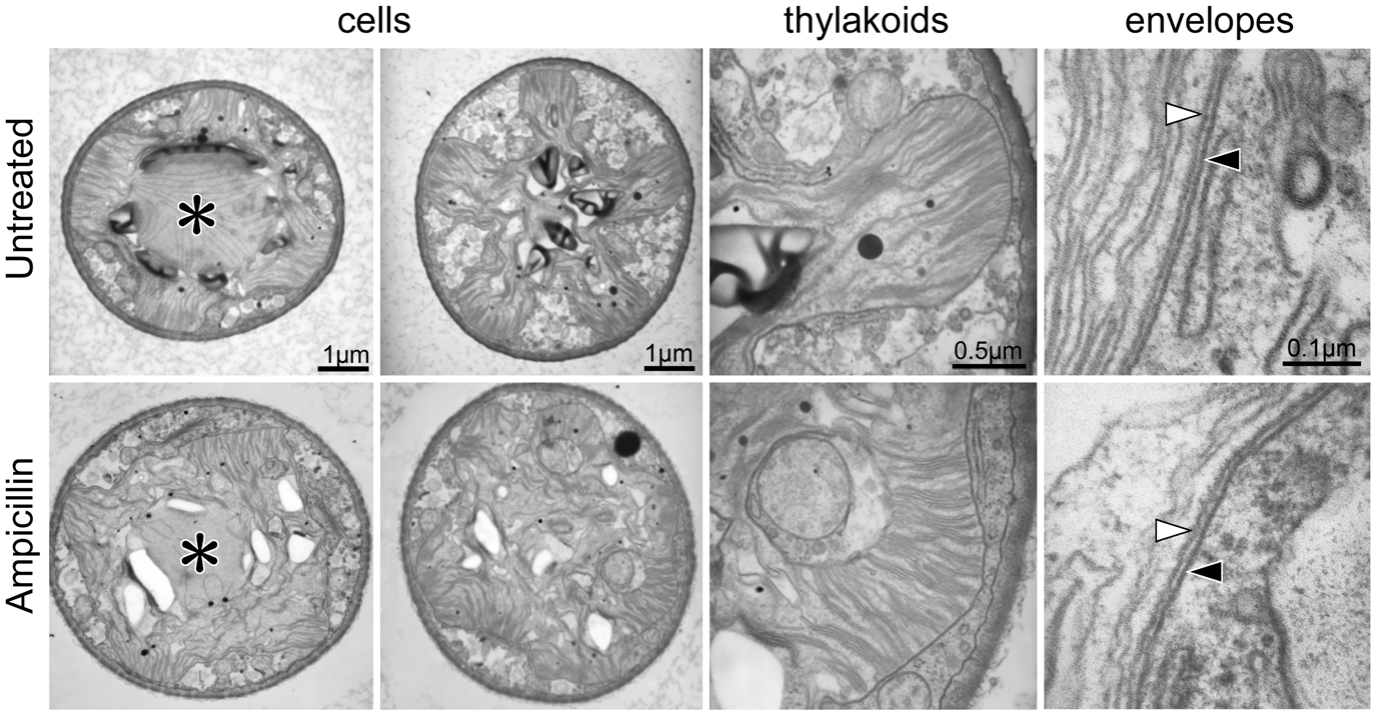
Electron micrographs of ampicillin-treated *Closterium* cells and of untreated controls. The magnification in the lower photos is the same as that in the upper photos. Pyrenoids surrounded by starch are indicated by asterisks. Black and white triangles indicate the outer and inner envelopes of chloroplasts, respectively.

To study the details in chloroplasts, ampicillin-treated cells were observed using electron microscopy. While star-shape chloroplasts with pyrenoids were observed in cells without ampicillin treatment, the shapes of the chloroplasts were irregular in the treated cells (Fig. 3). However, no obvious difference in the shapes of thylakoid membranes or of pyrenoids between the giant chloroplasts in treated cells and those of wild-type cells was observed. In treated or untreated cells, no rigid structures, similar to bacterial peptidoglycans, were detected between the inner and outer envelopes of chloroplasts in *C. psl*. complex.

**Figure 4:**
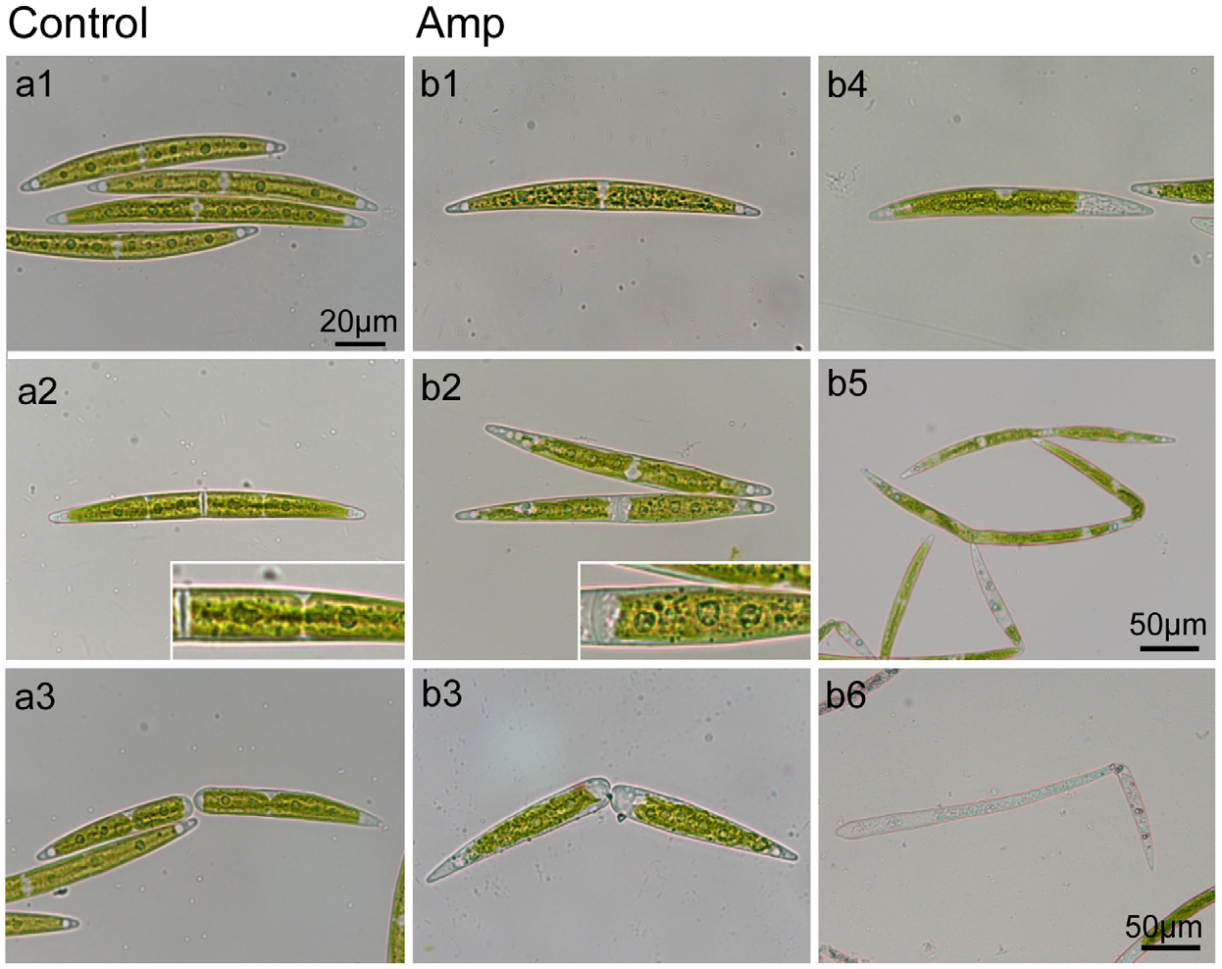
Representative micrographs of ampicillin-treated cells. For controls, dividing cells in the medium without ampicillin are shown (a1–3). When crescent-shaped cells (a1) enter the division process, chloroplasts started to divide (a2). After septum formation, two new semicells with two chloroplasts appeared (a3). In the medium with 100 µM ampicillin (b1–6), various types of cells were observed. There were normal crescent-shaped cells with two chloroplasts (b1), dividing cells without chloroplast division (b2), divided semicells with only one chloroplast (b3), crescent-shaped cells with one chloroplast (b4), long cells with irregular chloroplasts (b5) and dead cells (b6). The magnification in those photos without a scale bar is the same as that in a1.

**Figure 5:**
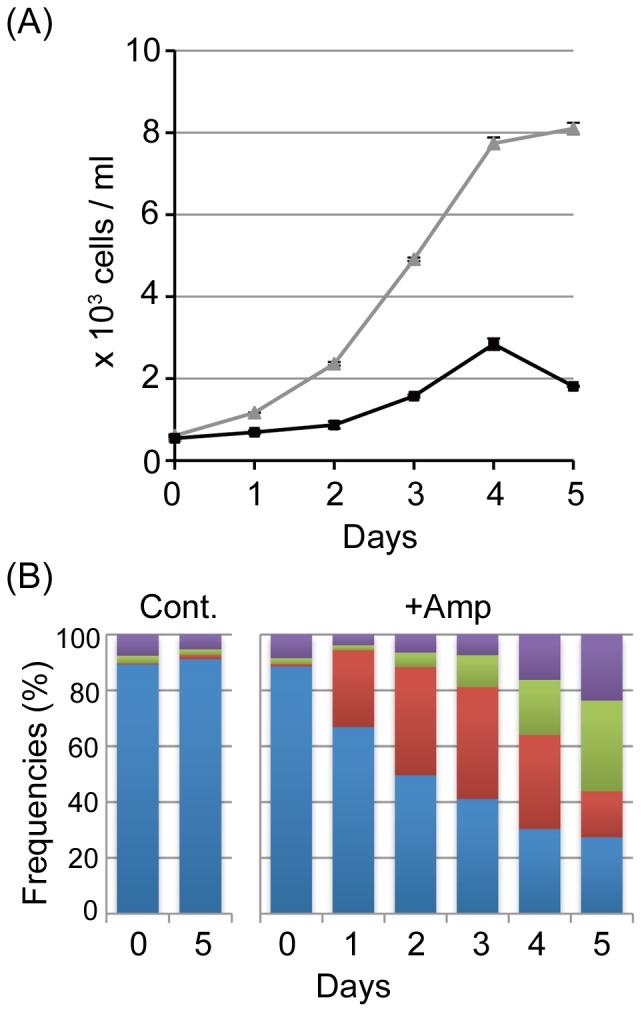
Effects of ampicillin on *Closterium* cell growth and division. (**A**) Growth curves of untreated cells (triangle) and of 100 µM ampicillin-treated cells (square). Measurements were performed in duplicate. (**B**) Changes in the frequencies of the four cell types: cells with two chloroplasts (Blue), cells with one chloroplast (Red), elongated cells with irregular chloroplasts (Green) and dead cells (purple). Measurements for untreated cells (Cont) at zero and five days and for ampicillin-treated cells (+Amp) are shown.

Treatment with ampicillin for five days resulted in appearance of long cells with huge chloroplasts and long dead cells (Fig. 4b5, b6). To uncover the reason(s) why these long cells appeared with ampicillin treatment, we measured cell numbers (Fig. 5A). In medium without ampicillin, *Closterium* cells multiplied 13-fold in five days. On the other hand, cells multiplied by 5.2-fold at four days after transfer to medium with ampicillin. This result suggested that ampicillin treatment interfered with cell growth. Next, we classified cells into four types based on the morphology and number of chloroplasts (Fig. 4, 5). The first and second types are crescent-shaped cells with two chloroplasts (Fig. 4a1, a3, b1–b2) and with only one chloroplast (Fig. 4b3–b4), respectively. The third and fourth are living long cells (Fig. 4b5) and dead cells (Fig. 4b6), respectively. Cell in excess of 1.5-times the length of a normal cell were recognized as long cells. The frequency of each cell type is shown in Figure 5B. In medium lacking ampicillin, approximately 90% of the cells were normal with two chloroplasts. In medium containing 100 µM ampicillin, the frequency of cells with only one chloroplast increased to 28% at one day after transfer to the ampicillin medium. This reached 40% at three days. At two days after transfer, the frequency of long cells was double that at day 0. The level of long cells increased during incubation and reached 32% at day five. The frequency of dead cells also increased gradually. These results showed that a cell with one chloroplast appeared first and then the length of the cell increased, suggesting that the huge chloroplast formed with ampicillin treatment were due to interference with cell division to create these long cells.

## Discussion

While ampicillin, D-cycloserine and fosfomycin inhibit chloroplast division in *C. psl.* complex, vancomycin and bacitracin had no obvious effect on chloroplast number (Fig. 2). Because these results are similar to those observed in the moss *P. patens* [5], with both species showing the same effects to a spectrum of antibiotics, they suggest that plastids in both organisms have a common ancestor. Presently, the reason for the specificity of the antibiotics is unclear. It may depend on enzymatic activity or infiltration through the cell wall, cell membrane, and/or plastid envelopes.

The effects of the antibiotics suggest that the peptidoglycan biosynthetic pathway is related to chloroplast division in the *C*. *psl.* complex, even though no wall-like structure has been detected in chloroplasts of the *C*. *psl.* complex (Fig. 3) similar to those in other organisms. A TBlastN search of the EST database suggested the possibility of genes encoding MurE, MurF, and PBP in the charophyte *Nitella hyalina*. If a charophycean algae genome is published in the future, we can evaluate whether *Mur* genes exist in these algal group, including *C. psl*. complex. The results in this paper suggest that, in the evolution of green algal lineage, the plastid peptidoglycan system remains in charophycean algae in addition to its independent loss in chlorophyte algae. At present, we cannot completely exclude the possibility that inhibition of chloroplast division was due to pleiotropic effects of antibiotics used and that Mur proteins work in other machinery related to chloroplast division but not to peptidoglycan biosynthesis. Whether plastid peptidoglycan exists in green plants remains unanswered. Experiments for peptidoglycan purification from *P. patens* or *C. psl.* complex and biochemical characterization using matrix-assisted laser desorption ionization (MALDI)-MS may be useful.

**Figure 6:**
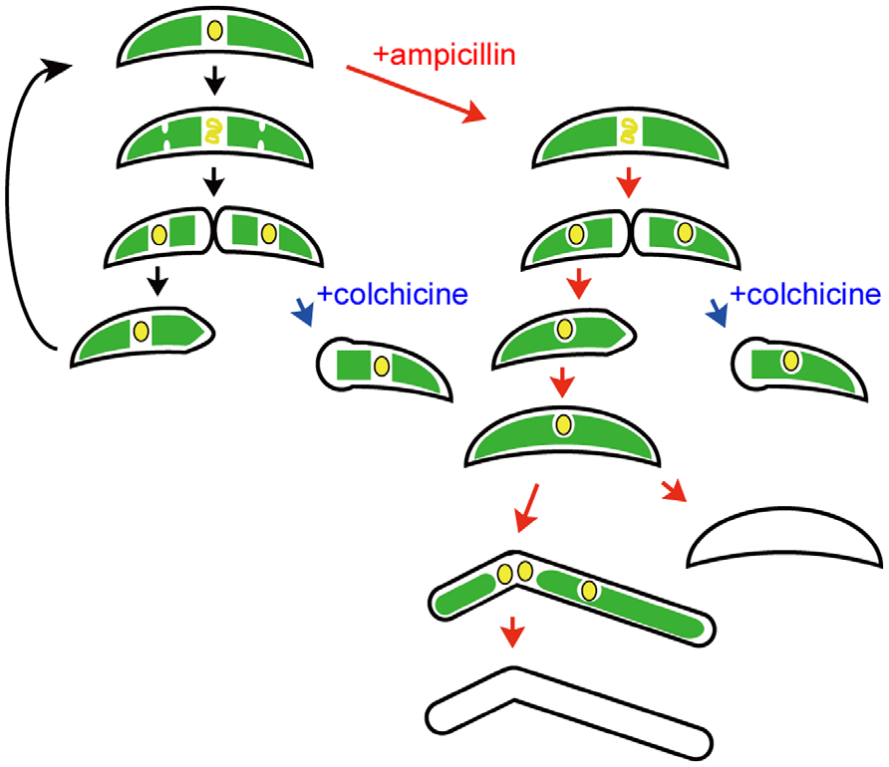
Morphological changes in *Closterium* cells treated with antibiotics. Without ampicillin (left panel), each of two chloroplasts divides before septum formation. After cell division, the daughter cell restores the crescent-shaped morphology with expansion of the new semicell. Colchicine inhibits elongation of the new semicells at 2 mM. With ampicillin (right panel), cells divide without chloroplast division to form semicells with only one chloroplast. Because cells can elongate without colchicine, crescent-shaped cells with only one chloroplast form. Then, long cells with several nuclei appear due to inhibition of cell division. During a long cultivation, cells die off.

Morphological changes in *Closterium* cells treated with antibiotics are shown schematically in Figure 6. The primary effect of antibiotic treatment appears to be inhibition of chloroplast division to generate semicells with one chloroplast. Experiments with colchicine suggested that these peptidoglycan-effecting antibiotics did not inhibit cell division in *C. psl*. complex, because we could observe many cells after cell division. Without colchicine, divided cells can elongate to form crescent-shaped cells, although the number of chloroplasts stays at one. Then, cell division is inhibited, probably by the huge chloroplast in the cell. Finally, elongation without cell division causes the appearance of long cells and subsequent cell death. Defects in growth and cell division were not observed in *P. patens* treated with antibiotics [5], [6]. While *P. patens* has about 50 chloroplasts per cell in normal conditions, only two chloroplasts exist within cells of the *C. psl*. complex. Thus, imperfections in chloroplast division may directly influence cell viability in *Closterium*.

## Materials and Methods

### Algal Materials and Antibiotics

*Closterium peracerosum–strigosum–littorale* complex strain NIES-67 was supplied by the National Institute for Environmental Studies, Ibaraki, Japan. Cells were usually grown in 100 mL Erlenmeyer flasks containing 25 mL of nitrogen-supplemented medium (C medium; http://mcc.nies.go.jp/top.jsp) on a rotary shaker (100 rpm) in a climate-controlled chamber at 24°C under a 16/8-h light/dark cycle or under continuous light (50–60 µmol photon m^−2^ s^−1^).

The antibiotics used were ampicillin sodium (Nacalai Tesque, Inc, Kyoto, Japan), fosfomycin sodium, D-cycloserine, bacitracin, and vancomycin (Wako Pure Chemical Industries, Ltd., Osaka, Japan). Each antibiotic was used at three or four concentrations in the preliminary experiments. The effects were the same among the conditions at concentrations of 100, 250, 500 and 750 µM for ampicillin and D-cycloserine, we used at the 100-µM concentrations. Because bacitracin showed no obvious effect at concentrations of 100, 250, and 500 µM, we used the condition at the 100-µM concentration. Fosfomycin was used at a concentration of 750 µM, because higher concentration was more effective. For vancomycin, we used a 100-µM concentration, because vancomycin affected cell viability at higher concentrations.

### Treatments with Antibiotics and Colchicine

Cells under a 16/8 h light/dark cycle were transferred into new medium with each antibiotic at 2 h after the light to dark shift. After 4–5 days cultivation, colchicine was added to the media to a final concentration of 2 mM. Because the frequency of cell division was stable after 4–5 days treatment, we used the cells at these days. Cells that divided in the dark period were observed at its end, because cells undergo cell division around 4 h after the beginning of the dark period [18]. Colchicine was from Wako Pure Chemical Industries. Bright-field cell images without fixation were recorded with a CCD camera (Nikon DXM1200 or Zeiss Axiocam) under a microscope (Olympus BX60 or Zeiss Axioskop 2 plus).

To remove ampicillin, ampicillin-treated cells were washed twice with C medium, and transferred into medium with 2 mM colchicine at 2 h after light to dark shift on day four. In the control experiment, with continuous ampicillin treatment, we added ampicillin in addition to colchicine. At the end of the dark period, the concentration of colchicine was increased to 4 mM. While colchicine at 2 mM inhibits the elongation of semicells without interfering with cell division of *C. psl.* complex, colchicine at 4 mM inhibits cell division in addition to the elongation of new semicells, [19]. Cells were observed at the end of the light period.

For continuous observation of ampicillin-treated cells, *Closterium* cells were grown under 24 h light conditions. The growth of *Closterium* cells was monitored by counting living cell numbers under a microscope every day after ampicillin treatment. At the same time, we classified cells into four types: cells with two chloroplasts, cells with one chloroplast, long cells with irregular chloroplasts, and dead cells. To determine the level of each cell type, we enumerated dead and living cells.

### Electron Microscopy

Cells cultured for five days in the presence of ampicillin were used. For electron microscopic observations, samples were fixed in 2% glutaraldehyde and 0.2% tannic acid buffered with 50 mM sodium cacodylate, and in a 1% osmium tetroxide aqueous solution containing 0.05% potassium hexacyanoferrate (II), dehydrated through an ethanol series, and embedded in Quetol-651 resin. Thin sections were cut and stained with uranyl acetate and lead citrate and then observed with a JEM-1200EX electron microscope (JEOL, Tokyo, Japan).

## References

[1] Archibald JM, Bullerwell CE (2012). Plastid origins..

[2] Steiner J, Löffelhardt W (2002). Protein import into cyanelles.. Trends Plant Sci.

[3] Takano H, Takechi K (2010). Plastid peptidoglycan.. Biochim Biophys Acta.

[4] Kasten B, Reski R (1997). b-lactam antibiotics inhibit chloroplast division in a Moss (Physcomitrella patens) but not in Tomato (Lycopersicon esculentum).. J Plant Physiol.

[5] Katayama N, Takano H, Sugiyama M, Takio S, Sakai A (2003). Effects of antibiotics that inhibit the bacterial peptidoglycan synthesis pathway on moss chloroplast division.. Plant Cell Physiol.

[6] Machida M, Takechi K, Chung SJ, Kuroiwa H, Takio S (2006). Genes for the peptidoglycan synthesis pathway are essential for chloroplast division in moss.. Proc Natl Acad Sci USA.

[7] Homi S, Takechi K, Tanidokoro K, Sato H, Takio S (2009). The peptidoglycan biosynthesis genes MurA and MraY are related to chloroplast division in the moss Physcomitrella patens.. Plant Cell Physiol.

[8] Banks JA, Nishiyama T, Hasebe M, Bowman JL, Gribskov M (2011). The Selaginella genome identifies genetic changes associated with the evolution of vascular plants.. Science.

[9] Izumi Y, Kuroki J, Nagafuji H, Lin X, Takano H (2008). Effects of antibiotics that inhibit bacterial peptidoglycan synthesis on plastid division in pteridophytes.. Cytologia.

[10] Matsuzaki M, Misumi O, Shin-I T, Maruyama S, Takahara M (2004). Genome sequence of the ultrasmall unicellular red alga *Cyanidioschyzon merolae* 10D.. Nature.

[11] Hedges SB, Blair JE, Venturi ML, Shoe JL (2004). A molecular timescale of eukaryote evolution and the rise of complex multicellular life.. BMC Evol Biol.

[12] Zimmer A, Lang D, Richardt S, Frank W, Reski R (2007). Dating the early evolution of plants: detection and molecular clock analyses of orthologs.. Mol Genet Genomics.

[13] Merchant SS, Prochnik SE, Vallon O, Harris EH, Karpowicz SJ (2007). The Chlamydomonas genome reveals the evolution of key animal and plant functions.. Science.

[14] Prochnik SE, Umen J, Nedelcu AM, Hallmann A, Miller SM (2010). Genomic analysis of organismal complexity in the multicellular green alga Volvox carteri.. Science.

[15] Wodniok S, Brinkmann H, Glöckner G, Heidel AJ, Philippe H (2011). Origin of land plants: Do conjugating green algae hold the key?. BMC Evol Biol.

[16] Sekimoto H, Tanabe Y, Tsuchikane Y, Shirosaki H, Fukuda H (2006). Gene expression profiling using cDNA microarray analysis of the sexual reproduction stage of the unicellular charophycean alga Closterium peracerosum-strigosum-littorale complex.. Plant Physiol.

[17] Iino M, Hashimoto H (2003). Intermediate features of cyanelle division of Cyanophora paradoxa (glaucocystophyta) between cyanobacterial and plastid division.. J Phycol.

[18] Hogetsu T, Shibaoka H (1978). The change of pattern in microfibril arrangement on the inner surface of the cell wall of Closterium acerosum during cell growth.. Planta.

[19] Hogetsu T, Shibaoka H (1978). Effects of colchicine on cell shape and on microfibril arrangement in the cell wall of Closterium acerosum.. Planta.

